# Influence of organic ammonium derivatives on the equilibria between NH_4_^+^, NO_2_^−^ and NO_3_^−^ ions in the Nistru River water

**DOI:** 10.1038/s41598-022-17568-3

**Published:** 2022-08-05

**Authors:** Petru Spataru

**Affiliations:** Institute of Chemistry of the Republic of Moldova, 3 Academiei str, Chisinau, MD-2028 Republic of Moldova

**Keywords:** Ecology, Environmental sciences, Chemistry

## Abstract

The toxic effects of ammonium derivatives in the river water depend dramatically on their natural or synthetic origins and on their chemical structures. It has been proved that 1-naphtylamine (1-NA) and diphenylamine (DPA) breaking impact on the ammonium oxidation and especially on nitrite ions oxidation processes in natural waters is associated with its toxicity. The NH_4_^+^ oxidation process slows down for about five days and ten days in river water samples with 0.5 mg/L DPA and corresponding 0.5 mg/L 1-NA. The NO_2_^−^ oxidation delay in model samples of river water with 0.025 and 0.05 mg/L 1-NA, is four days and 35 days in the one with 0.5 mg/L 1-NA. For the sample with 0.05 mg/L DPA the delay of the NO_2_^−^ oxidation is approximately of six days and 25 days for sample with 0.5 mg/L, DPA. The laboratory simulations have revealed: (1) absorption–desorption, the micro biotic reaction to the instantaneous increase of the concentration of ammonium ion in the river water (so-called shock/stress effect) and (2) the NH_4_^+^ increase stimulated by a certain (0.05 mg/L) concentration of 1-NA.The diethylamine (DEA) decomposition leads to increasing with approximately 3.8 mg/L NH_4_^+^ in river water samples of 20.0 mg/L DEA.

## Introduction

Ammonium ions exported from water basins are a product largely derived from the degradation of organic matter of protein origin, which manifests a selective toxic effect^1–3^. Similar to carbon dioxide and methane, it is a final product of living organisms and substrates of the combustion/fermentation processes.

Ammonium and nitrite ions are some of the most dangerous for fish in natural water pools. Only about 0.2 mg/L ammonium has a negative impact on the development of fish larvae^4^. Nitrite ion converts hemoglobin to methemoglobin, producing vasodilation and damaging vitamin A stores. In addition to these direct toxic effects, nitrites and nitrates interact with simple, secondary, and tertiary amines, nitrogen oxides, and quaternary ammonium compounds, with the formation of nitrosamines, toxic substances with carcinogenic potential^5^.

The influence of the decomposition of the excrement, a product of the activity of the animals and man, must be taken into account as a source of NH_4_^+^ in the wastewater analysis. In the Republic of Moldova our physicochemical analyzes show that urban wastewaters contain about 20–60 mg/L of NH_4_^+^ at the entrance to the WWTPs, while rural domestic wastewaters contain over 100 mg/L of NH_4_^+^. Wastewater from animal complexes usually exceeds 1600 mg/L and sometimes even up to 5600 mg/L of NH_4_^+^. In more developed countries this indicator is lower^6–8^.

The presence of ammonium in natural water stimulates an increase in algae and heterotrophic (e.g. *Pseudomonas putida*), autotrophic (AOB, *Nitrosomonas and Nitrosospira*; AOA, *Nitrososphaerea, Nitrosopumilus*) bacterial activity^9–13^. As a result of the degradation and decarboxylation of protein amino acids^14–16^, the aquatic environment contains a large variety of organic ammonium derivatives, in which hydrogen atoms are partially or totally replaced by different radicals. A general process for the natural formation of the amines in aquatic basins can be presented by a simple scheme:

$$RCH(NH_{2} ) - COOH\mathop{\longrightarrow}\limits^{decarboxylase}RCH_{2} - NH_{2} + CO_{2}$$

Besides amines of natural origin, amines produced by industrial chemical synthesis are often present as well in water basins. The amines of industrial/synthetic origin, especially aromatic ones, are more toxic and more difficult to decompose in the aquatic environment compared to natural derivatives, creating in WWTP emerging pollutants of concern. Pollution becomes even more dangerous if the industrial azo-dyes in an anaerobic environment are reduced to aromatic amines^17–22^. It should be taken into account also that the addition of ammonium stimulates supplementary sewage treatment processes^23–25^. On the other hand, as a result of discharging of insufficiently treated wastewater into rivers, an additional quantity of CaCO_3_ leads to the reactivation of cationic organic pollutants with a negative impact on the self-purification process and in particular for the oxidation of NH_4_^+^ and NO_2_^−^ ions to nitrate, e.g. nitrification. The breaking phenomenon of nitrification in the presence of CaCO_3_ is characteristic of the river sections polluted with urban wastewater, where the cationic surface-active substances (SAS) and other emerging substances are contained^26–29^. Consequently, a previously developed model of the arrangement of SAS on the surface of calcium carbonate nanoparticles has been taken into consideration^30,31^. The model is based on (*a*) the fixation of the anionic portion of the molecule and further decomposition of the SAS-Ct·SAS-An complex and (*b*) on the change of the cationic part in the solution of water increasing the breaking activity of ammonium derivatives^27–29^).

The investigated samples containing SAS-Ct and SAS-An were analyzed in aqueous solutions with and without CaCO_3_ by the UV spectroscopy method^28^. The UV spectra show the decomposition of the SAS-Ct · SAS-An complex and the activity of suppressing the oxidation of NH_4_^+^ and NO_2_^−^ ions when adding calcium carbonate to river water laboratory models. Thus, the surface of the calcium carbonate in the absence of ammonium ion binds SAS-An only, however upon adding an aqueous solution of NH_4_^+^, the surface of the CaCO_3_ particles may adsorb both the anionic and cationic SAS.

Anilines being organic derivatives, in which hydrogen atoms from the aromatic cycle are replaced by organic radicals, have a toxic influence on living organisms in aquatic basins^22,32,33^. The studied aromatic amines by analogy could be considered as well toxic to aquatic microorganisms.

Just as amino acids are a food for many living organisms, so ammonia or ammonium (NH_4_^+^), which is protonated ammonia, can be a source of building material and energy for a number of consumers for whom the model is primarily initiated. Thus, the addition of a definite amount of ammonia nitrogen triggers the stimulation processes in which a priori there are certain amounts of NH_4_^+^, NO_2_^−^ and NO_3_^−^ ions as well as organisms that can accept these soluble species of nitrogen as a source of their existence.

A dynamical balancing and consumption process influences the hydro-microbial component, composition and concentration of organic matter in natural water after adding additional amount of ammonia nitrogen. River or lake water as a rule also has dissolved oxygen that participates during laboratory modeling in above mentioned processes. Our previously obtained data^34^ show that there is a close correlation between the initial added amount of NH_4_^+^ in a river or lake water sample and the maximum concentration of nitrite ion as an intermediate species in the natural water model.

The nitrification is a basic process in our models in which there could be many competing processes during the laboratory simulations. There was a close correlation between the initial ammonia nitrogen dose and the maximum nitrite ion concentration for the river or lake water sample models, in which the effective initial concentrations was equal from 2 to 14 mg/L of NH_4_^+^. In our experiments the control tests of the river or lake water samples had the same conditions except for the absence of the act of initiation by ammonium. However, when estimating the impact of any pollutant or of certain amounts of the substance of some quality, the control sample was the one in which only the dose of NH_4_^+^ was added to the laboratory simulation^34^.

The study of the behavior of stable nitrogen forms (NH_4_^+^, NO_2_^−^, NO_3_^−^) in natural water basins is crucial in discovering new insights regarding the environmental matrix^17,22^. The investigation of the dynamics of these nitrogen species concentrations in laboratory models for a large number of river and lake waters leads to the conclusion that changes in their concentrations can serve as sensitive indicators of the composition and concentration of the organic matter and its transformations in this environment^27–29,34–36^. The ammonium and its environmental amine derivatives are physiological products released through a wide range of bacterial catalytic systems^3,9–16,18,19,21^ as well as through natural redox processes. The increased mutagenicity found in bacterial strains indicates the presence of nitroarenes and/or aromatic amines (methyl parathion, 1-naphthylamine, and N-phenyl-2-naphthylamine) in the underwater sediment of the Elbe River Basin^37^. By analogy it could be anticipated a high sensitivity effect for 1-NA in our laboratory simulations of the NH_4_^+^ and NO_2_^−^ oxidation processes.

Determining the influence of the abovementioned ammonium derivatives on the chemical equilibria between stable nitrogen species (NH_4_^+^, NO_2_^−^, NO_3_^−^) along with the involved redox processes, was the main goal of this paper.

## Materials and methods

To carry out the laboratory modeling, a water sample was taken from the Vadul-lui-Voda section of the Nistru River at a distance of 5 m from the river bank where the water depth was over 2 m, on May 16th 2019. The temperature of water was 15.8 °C. The average parameters of Eh, pH and rH_2_, measured three times in a day, were equal to 0.33 V, 8.15 and 27.7, respectively. The rH_2_ value indicated a state of water between slightly polluted and pure. The average value of the dissolved oxygen was equal to 10.7 mg/L. The river water was left overnight for sedimentation of suspended organic-mineral particles and then model samples were prepared in a room specially designed for laboratory modeling in natural light conditions, but protected from direct sunlight. The average COD and BOD_5_ in the water sample from the Nistru River were 18.3 and 2.81 mgO/L respectively. Concentrations of natural amines in river waters were between 10 and 200 µg N/L^38^, it is much lower compared to those of natural origin (e.g. DEA) used in models. No sources of amine pollution of synthetic origin are known on the entire portion of the Nistru River belonging to the Republic of Moldova. The nitrogen species concentrations in the river water sample models were equal to 0.12 mg/L NH_4_^+^, 0.028 mg/L NO_2_^−^, and 4.5 mg/L NO_3_^−^. In three liters of river water sample 9 mL of solution with a concentration of 1 mg/mL NH_4_^+^ was added to obtain a concentration of ≈3 mg/L NH_4_^+^ ions and to another series of samples 18 mL of the same solution in a sample river water was added to obtain an additional number of models with 6 mg/L NH_4_^+^ initial concentration. Only NH_4_Cl solution (1 mg/mL) was added in the reference sample, while the working samples contained also amines (1-NA, DPA and DEA). Samples with 1-NA of 0.025 mg/L (0.5 MAC of 1-NA), 0.05 mg/L (1.0 MAC of 1-NA), 0.1 mg/L (2.0 MAC of 1-NA), 0.25 mg/L (5.0 MAC of 1-NA) and 0.5 mg/L (10.0 MAC of 1-NA) were prepared using solution with chemically pure 1-naphthylamine. Both amines of synthetic origin (DPA) and those resulting from natural processes (DEA) were investigated at a lower, maximum admissible concentration (MAC) and higher concentration of 10 MAC. Samples were prepared respecting the minimum recommended amount of water in glass vessels^39^. The experiment consisted on testing of nitrogen species NH_4_^+^, NO_2_^−^and NO_3_^−^ after one day, two, and so on, at the same time of each day (in our case starting at 10 a.m.). After each test, the samples were thoroughly stirred. Analysis of natural waters was accomplished according to the ISO methods^40–44^. The ISO physicochemical methods, cited in references, were used to monitor the transformation of the nitrogen species NH_4_^+^, NO_2_^−^, NO_3_^−^ and pH to the influence of the microbial enzymatic system, which in turn is a function of the chemical composition and in particular of organic matter in natural waters (river, lake, sewage basin, etc.). The pH tests showed a line parallel to the horizontal axis. The chemicals used in the experiment were of purity grade “puriss”. Sample testing was accomplished using the HACH DR/2500 Spectrophotometer and UV spectroscopy using Perkin Elmer Lambda 25.

### Consent for publication

I agreed to publication in the Scientific Reports journal.

## Results and discussions

**Figure 1:**
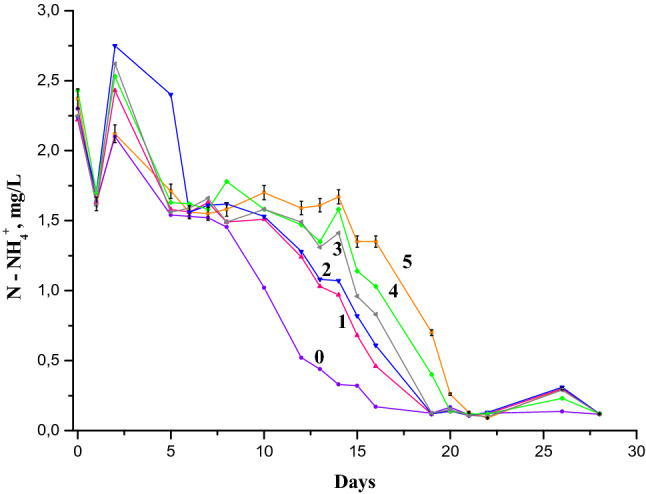
Dynamics of ammonium ions concentration in the Nistru River water containing 3 mg/L NH_4_^+^ in the presence of 1-naphthylamine^*^: (0) 0 mg/L, reference sample; (1) 0.025 mg/L (0.5 MAC 1-NA); (2) 0.05 mg/L (1.0 MAC 1-NA); (3) 0.1 mg/L (2.0 MAC 1-NA); (4) 0.25 mg/L (5 MAC 1-NA); (5) 0.5 mg/L (10 MAC 1-NA). *The indicated chemical analysis tests were obtained through three replicates. Other curves on this figure contain the same uncertainties, which for simplicity are not indicated.

**Figure 2:**
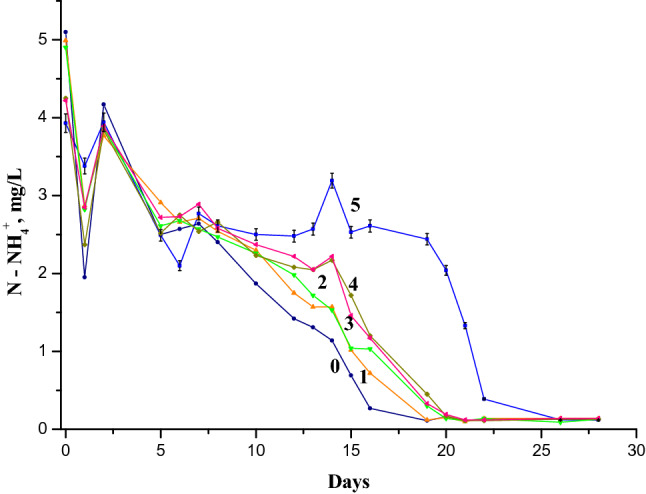
Dynamics of ammonium ions concentration in the Nistru River water containing 6 mg/L NH_4_^+^ in the presence of 1-naphthylamine: (0) 0 mg/L, reference sample; (1) 0.025 mg/L (0.5 MAC of 1-NA); (2) 0.05 mg/L (1.0 MAC of 1-NA); (3) 0.1 mg/L (2.0 MAC of 1-NA); (4) 0.25 mg/L (5 MAC of 1-NA); (5) 0.5 mg/L (10 MAC of 1-NA).

The ammonium ion concentration decreases dramatically during the first day, but then it increases on the second day after the initiation of laboratory simulations (Figs. 1 and 2), which serves as a proof of the absorption–desorption processes by biota.

The changes in the first three days of the experiment described in Figs. 1 and 2 were found in the same way in the models with water from other rivers and lakes starting from the concentration of 1 mg/L NH_4_^+^ without other additions. No tests were performed at concentrations lower than 1 mg/L NH_4_^+^ due to the errors that could occur at such low concentrations. As it can be seen from Figs. 1 and 2, the concentration of 1-NA has an impact especially at MAC level, 0.05 mg/L. The decreasing effect on the first day and increasing the concentration of ammonium ions on the second day were analyzed within a large range of 1-NA concentrations: 0.025, 0.05, 0.1, 0.25 and 0.5 mg/L, or 0.5; 1; 2; 5 and 10 MACs, due to its special impact^34^ at the instantaneous increase of NH_4_^+^ in the aquatic environment, which was described below.

**Figure 3:**
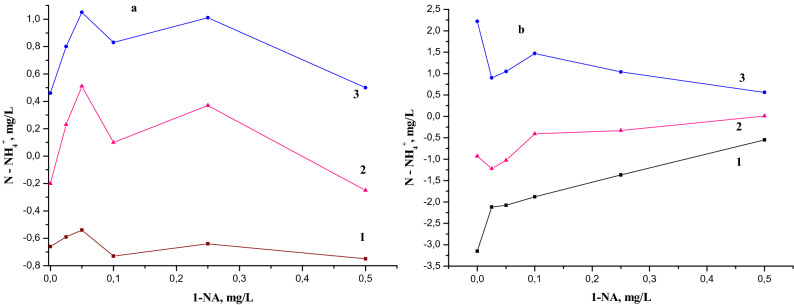
(**a**) The following differences of ammonium ion concentrations: (1) (C1-C0); (2) (C2-C0); (3) (C2-C1) in function of 1-NA concentration with initial 3 mg/L NH_4_^+^. (**b**) The same functions with initial 6 mg/L NH_4_^+^.

The analysis of the NH_4_^+^ concentration difference (in mg/L) was measured starting from the time of the laboratory incubation (initiation). Three time periods were considered: (1) the difference between the values of the initial concentration (C0) and that obtained after one day (C1), i.e. (C1-C0); (2) the difference between the values obtained after two days of experience and the initial ones, (C2-C0); (3) the difference between the values obtained after two days of experience (C2) and those after one day of experience (C1), (C2-C1) as a function of the 1-NA concentrations when the initial ammonium ion concentration was 3 mg/L (Fig. 3).

One can observe from Fig. 1 and Table 1 that the total mineral nitrogen (e.g., the total sum of the NH_4_^+^, NO_2_^−^ and NO_3_^−^) concentration is increasing during the period of nitrogen fixation (starting on second day and continuing on fifth day). Thus, the increase of ammonium nitrogen is not due to other soluble nitrogen species sources of the river water. A similar growth in the ammonium ion concentration was detected in the river water experiment within laboratory models with the initial concentration of 2 mg/L NH_4_^+^ for the same 0.05 mg/L concentration of 1-NA^34,35^.

**Table 1:** Total mineral nitrogen concentration in river water experiments.

Test time	Reference test, initial 3 mg/L NH_4_^+^	0.05 mg/L1-NA and initial 3 mg/L NH_4_^+^	Reference test, initial 6 mg/L NH_4_^+^	0.05 mg/L1-NA and initial 6 mg/L NH_4_^+^
0 day	3.33 ± 0.356	3.31 ± 0.356	6.13 ± 0.658	5.93 ± 0.637
1 day	2.86 ± 0.305	2.67 ± 0.288	2.93 ± 0.315	3.98 ± 0.427
2 days	2.72 ± 0.292	3.14 ± 0.337	4.53 ± 0.487	4.18 ± 0.449
5 days	2.60 ± 0.279	3.29 ± 0.354	3.25 ± 0.349	3.32 ± 0.357
6 days	2.56 ± 0.275	2.65 ± 0.285	3.58 ± 0.385	3.46 ± 0.372
7 days	2.52 ± 0.271	2.61 ± 0.281	3.75 ± 0.403	3.54 ± 0.381

A large peak on the curves of the differences of NH_4_^+^ concentrations depending on the concentration of 1-naphthylamine, between the first and the fifth day was observed in the series with the initial concentration of 3 mg/L NH_4_^+^ (Fig. 3a). This peak is maximal and more pronounced for the sample with 0.05 mg/L of 1-NA concentration (Fig. 3a and Table 1), compared to other 1-NA concentrations of 0.5, 0.25, 0.1, and 0.025 mg/L. Therefore, it can be assumed the concentration of 0.05 mg/L 1-NA stimulates nitrogen-fixing species in river waters. In our previous experiments, the concentration of ammonia nitrogen increased by more than 50% of the initial NH_4_^+^ concentration in the sample with a similar concentration of 1-NA after three days of the experiment^34^. Also, the NH_4_^+^ concentration increase did not stop after three days and continued for about ten days for the natural water samples in the spring period^34^.

In natural waters, the changes caused by adding NH_4_^+^ lead to stable biochemical equilibrium throughout the entire experiment involving microbial populations with nitrogen fixation properties. Some microbial populations use ammonium nitrogen compounds for growing, while others use them for maintaining their lives through the redox process of nitrogen species^26,45–47^. The N-fixation and oxidation of ammonium and nitrite ions and as well redox processes of nitrogen-containing compounds take place in the same aquatic environment, influencing the change in microbial populations. During the second day after incubation, the NH_4_^+^ concentration exceeded the initial value, then decreased (Fig. 3) for the samples of 0.25, 0.1, 0.05 and 0.025 mg/L, indicating to a drop of the ammonium concentration due to potential sorption and redox processes. There was a decrease of some in the concentration of NO_2_^−^ and NO_3_^−^ during this period. This change also occurred in the reference sample. The diminution in ammonium ion accumulation and the increase of the redox processes were more significant after the third day of the experiment (Figs. 1 and 2) leading to a decrease in ammonium ions concentration for all the samples. On the sixth day, the rate of the NH_4_^+^ oxidation process exceeded that of ammonium accumulation. The differences of ammonium ion concentrations as a function of the 1-NA concentration (Fig. 3a) suggest that the nitrogen fixation is still continuing.

The analysis of the natural water model behavior as a function of 1-NA concentration at initial concentration of 6 mg/L NH_4_^+^ was performed. Three sets of model experiments were selected: (1) the difference between the values of the initial concentrations (C0) and those obtained after one day (C1) of laboratory simulations, (C1-C0); (2) the difference between the values obtained after two days of experience (C2) and those after one day (C1), (C2-C1); (3) the difference between the values after two days of experience and initial values; (C2-C0) (Fig. 3b). From the calculations made it was possible to observe that in the first two days at the concentration of 6 mg/L NH_4_^+^, the 1-NA concentration has a well-defined impact. The values of significant NH_4_^+^ concentration growth due to the presence of 0.05 mg/L 1-NA in the 3 mg/L NH_4_^+^ samples were not typical for those with the initial concentration of 6 mg/L NH_4_^+^. The statistical processing showed that this sorption/desorption phenomenon is close to zero at the concentration value of 0.70 (± 0.035) mg/L 1-naphthylamine. Therefore, the value of 1-NA concentration at which it is predicted to completely stop the processes connected to the biochemical production and sorption of the NH_4_^+^ due to its toxicity, is equal about to 0.70 mg/L. One can assume that the decrease in ammonium concentration on the first day after the initiation and its increase on the second day, caused by aquatic microorganisms, could be called the micro-biotic reaction to pollution with ammonium (MBRA). The increase in the amount of ammonium ions in water samples leads to the shift of biochemical equilibria in the direction of ammonia nitrogen desorption. Exceeding the concentration of NH_4_^+^in water during the second day of the experiment the addition of 0.05 mg/L 1-NA, containing only 3 mg/L NH_4_^+^ in initial concentration, leads to the assumption of N-fixation and/or mineralization (see Figs. 1, 3 and Table 1). Indeed, the concentration of ammonium ions in this treatment increases starting from the second day compared to the its initial concentration, while in the control treatment on the same day its concentration decreases, compared with starting day value. The change in the ammonium ion concentration during the second day in the experiment with 6 mg/L of NH_4_^+^ as a starting concentration both treated with and without 0.05 mg/L 1-NA was similar (Table 1). Therefore, the “experimental” increase of the NH_4_^+^ initial concentration from 3 to 6 mg/L leads to the suppression of nitrogen fixation processes.

The increase of C1-C0, C2-C1 and C2-C0 parameters and the concentration of total mineral nitrogen in water (Fig. 3a) for 3 mg/L NH_4_^+^, contrary to the case of 6 mg/LNH_4_^+^ (Fig. 3b) shows that the presumed N-fixation could only occur in a lower concentration of ammonium ions at the same concentration of 0.05 mg/L of 1-NA. The registered decrease of the total nitrogen concentration in the model with 3 mg/L of initial NH_4_^+^ during the 6th and 7th days and the absence of this effect in the model with 6 mg/L of initial NH_4_^+^ leads to the supposition that the bacterial plankton responsible for the nitrogen absorption is suppressed at higher concentration of ammonium ions. This suppressive effect has been also described by other researchers^48–52^.

Nitrate ion converting by bacteria directly into ammonia is the dissimilatory nitrate reduction to ammonium (DNRA) process more common in soil, wetlands and lake sediments^53–55^. Nevertheless, it looks similar to the special model, e.g. conditions that would cause an insignificant oxygen deficit, where the aquatic microorganisms take advantage of the possibility of using oxygen from nitrate to synthesize directly ammonium ions without intermediate nitrite ions. Nitrite ion is one of the most toxic components for the aquatic environment. Definitely, the NO_3_^−^ → NH_4_^+^ redox transformation excluding the formation of intermediate NO_2_^−^ ions is most convenient both energetically and environmentally^56–58^.

**Figure 4:**
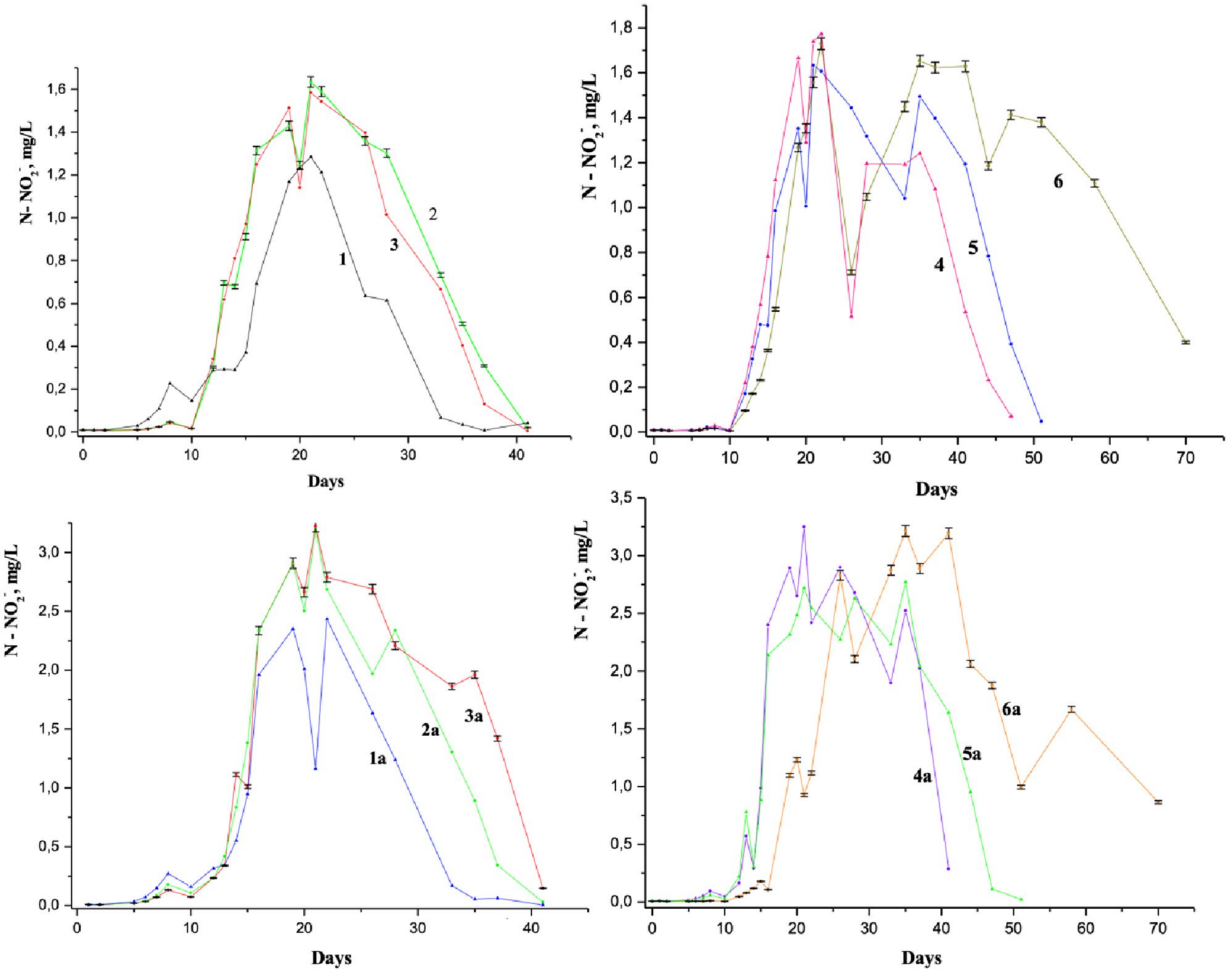
Dynamics of the nitrite ions concentration in the Nistru River water (Vadul-lui-Voda section), containing: 3 mg/L NH_4_^+^(plots from above) (1—the reference sample, 2—with 0.025 mg/L 1-NA, 3—with 0.05 mg/L 1-NA, 4—with 0.1 mg/L 1-NA, 5—with 0.25 mg/L 1-NA, 6—with 0. 5 mg/L 1-NA) and 6 mg/L NH_4_^+^ (plots from the bottom) (1a—the reference sample, 2a—with 0.025 mg/L 1-NA, 3a—with 0.05 mg/L 1-NA, 4a—with 0.1 mg/L 1-NA, 5a—with 0.25 mg/L 1-NA, 6a—with 0. 5 mg/L of 1-naphtylamine) initially.

Dynamics of NH_4_^+^ concentration in Figs. 1 and 2 and of NO_2_^−^ concentration in Fig. 4 and their comparative analysis point out the influence of 1-NA concentration on the river water self-purification process. Figures 1, 2, 4 show that at its equal concentrations of 1-NA and even lower than 1 MAC of 1-NA, the ammonium oxidation occurs slower than in the reference sample. If the concentrations of 1-NA exceed the MAC values, the breaking ammonium oxidation is more pronounced. In other words, the NO_2_^−^ oxidation into NO_3_^−^ proceeds more slowly than the formation of NO_2_^−^ by NH_4_^+^ oxidation leading to the increase of NO_2_^−^ concentration^59,60^. At the same time, the NO_2_^−^ accumulation becomes gradually more pronounced as the concentration of 1-NA increases.

By comparing the dynamics of the ammonium ion concentration with those of nitrite ion formation, one can observe that the natural aquatic environment shows a high sensitivity to the presence of 1-naphthylamine even at its much lower concentrations than MAC. On the other hand, at the equal concentrations of the components in the Figs. 1, 2, 3, 4 the dynamics of the NH_4_^+^ and NO_2_^−^ displays an equilibrium between these two ions in our models, namely the converting of ammonium ions into nitrite and vice versa. There are no doubts that the transformation of various forms of organic carbon in a complicated equilibrium mechanism in natural waters is related to the oxygen consumption from nitrate and nitrite. Figure 4 shows that the oxidation of ammonia and intermediate forms of nitrogen oxidation leads finally to the nitrate formation in the river waters. Thus, the decrease in the NO_2_^−^ concentration at the 20th day is noticeable within the laboratory simulations with 1-naphthylamine in both 3 mg/L and 6 mg/L NH_4_^+^ initially containing samples. Generally, the increase of ammonium ions and 1-naphthylamine concentrations causes a significant influence in the natural water biochemical equilibria, leading to the increase in the duration of redox intermediate processes of formation and especially oxidation of NO_2_^−^ (Fig. 4). Previous physiochemical and microbiological research has been performed in laboratory models with amines in which the dynamics of nitrite ion concentration has been compared with numerical increase of the heterotrophic populations. Our earlier investigation showed that the dynamics nitrite concentration in these laboratory models indicates a synchronized increase in of NO_2_^−^ and heterotrophic and autotrophic development^34,35,61^.

**Figure 5:**
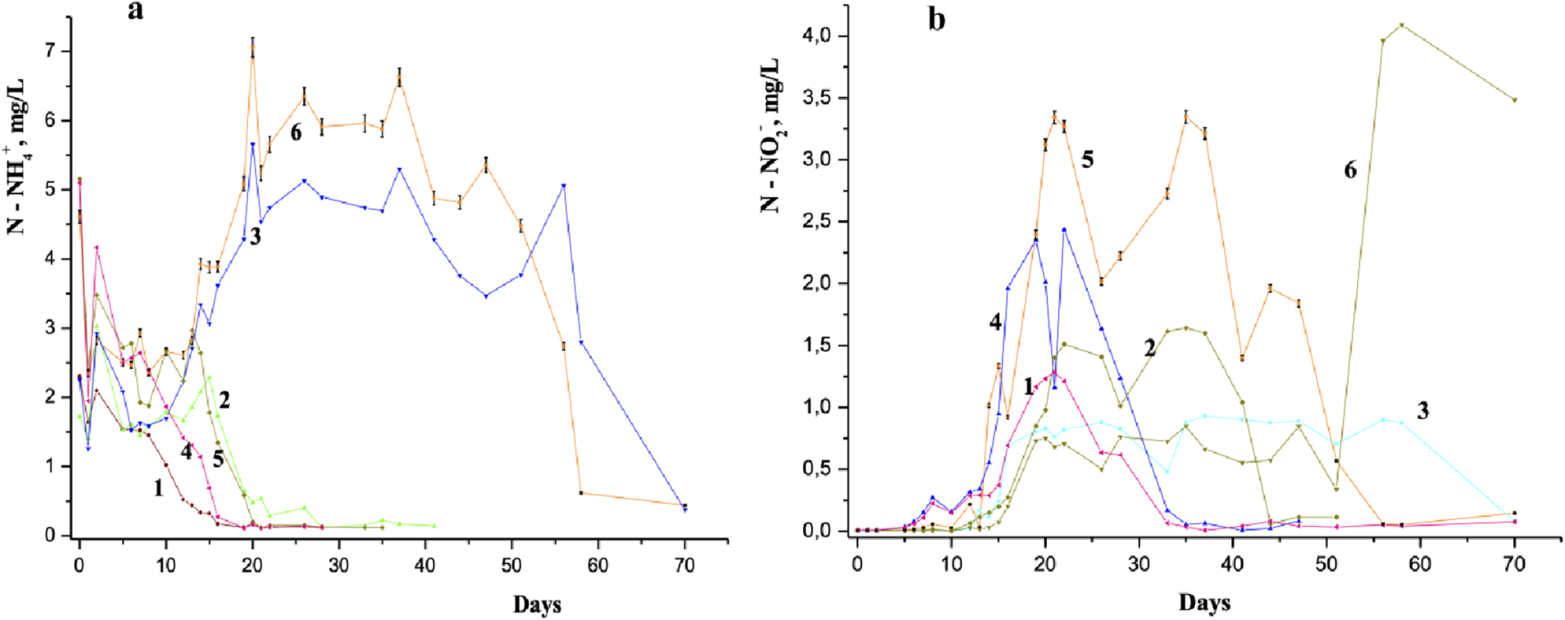
(**a**) Dynamics of the ammonium ion concentration in the Nistru River (Vadul-lui-Voda section) water on the modeling with 3 mg/L NH_4_^+^ (1—the reference sample, 2—with 2.0 mg/L, 3—with 20.0 mg/L DEA) and 6 mg/L NH_4_^+^ (4—the reference sample, 5—with 2.0 mg/L, 6—with 20.0 mg/L DEA)initially. (**b**) Dynamics of the nitrite ion concentration in the Nistru River (Vadul-lui-Voda section) water on the modeling with 3 mg/L NH_4_^+^ (1—the reference sample, 2—with 2.0 mg/L, 3—with 20.0 mg/L DEA) and 6 mg/L NH_4_^+^ (4—the reference sample, 5—with 2.0 mg/L, 6—with 20.0 mg/L DEA) initially.

The study of the equilibria between NH_4_^+^, NO_2_^−^ and NO_3_^−^ in natural waters in the presence of diethylamine (DEA) is relevant not due to its presence in the industrial waste but also because DEA it is assimilated by aquatic microorganisms. Our simulations show a completely different picture of the biochemical and redox equilibria in the presence of DEA (Fig. 5). First of all, it should be mentioned the large difference between the MAC values of the DEA samples, compared to 1-NA ones (almost larger by two orders). Samples with a relatively low concentration of 1-NA (between 0.025 and 0.5 mg/L) and those containing large amounts of DEA (between 2.0 and 20.0 mg/L) correspond to the same MAC values (between 0.5 and 10 respectively).

The decomposition, oxidation, and assimilation of DEA occurs naturally much easier compared to organic ammonium derivatives of synthetic origin and especially the aromatic ones. For 1-NA, the NH_4_^+^ oxidation is delayed during the first days of experiments (Figs. 4 and 5) comparing with the reference sample, even if after this time the NO_2_^−^ concentration in analyzed models is larger compared to the reference one. Our guess is that this oxidation delay comes from the effect of breaking NO_2_^−^ oxidation. In the case of DEA (Fig. 5), there is a delay of both the oxidation of NH_4_^+^ and the subsequent oxidation of NO_2_^−^ compared to the reference sample. Figure 5 shows that the adding of low DEA concentration (2.0 mg/L) leads to an increase of the nitrite amount and therefore to the greater rate of its formation, while the large content of DEA (20 mg/L) causes the decrease of the NO_2_^−^ amount both in 3 and 6 mg/L NH_4_^+^ containing samples. The concentration of 2.0 mg/L of DEA causes an enzymatic activation in which most of organic carbon species including DEA carbon radicals are present in the river water samples, without affecting too much the redox environment^21^. The increase of DEA concentration up to 20 mg/L leads to the growth of the amine nitrogen participation into the updated oxidation/reduction mechanism, which involves its converting in the ammonium form. This is due to the fact that the breakdown of this amine leads to the formation of significant amount of organic carbon with a high reducing activity, which contributes to the consumption of dissolved oxygen. The BOD_5_ (2.81 mgO/L) of the river water sample compared to the average concentration of dissolved oxygen (10.7 mgO/L) in this case can be considered as an environmental index that is in certain equilibrium in the process of oxygen consumption and recovery. Most of this oxygen deficiency has an impact on samples with 6 mg/L NH_4_^+^. The oxygen concentration in the „initial” waters, without adding anything, was approximately 10–11 mg/L. The reference sample containing 3 mg/L consumes 9.3 mg/L oxygen and 18.7 mg / L O_2_ the one with 6 mg/L NH_4_^+^ if the oxidation of ammoniacal nitrogen takes place up to NO_2_^−^. Biochemical oxygen demand in the sample with 3 mg/L and 2 mg/L DEA is close to the reference sample with 6 mg/L NH_4_^+^, which exceeds the concentration of dissolved oxygen by approx. 70–85%. Biochemical oxygen demand in the model sample with 6 mg/L and 2 mg/L DEA exceeds the concentration of dissolved oxygen by more than 2.5 times. The process of recovering dissolved oxygen takes place permanently and therefore, the samples described above are less affected by the lack of oxygen. However, both the model samples, containing 20 mg/L DEA with 3 mg/L NH_4_^+^ and especially with 6 mg/L NH_4_^+^, have an oxygen deficiency, which causes the decrease of the activity and the numerical value of the microbial populations with the activity of oxidation and assimilation of organic carbon and ammonia nitrogen. This can lead to a decrease in populations of microorganisms related to oxidation and assimilation of carbon and nitrogen with a low oxidation state (with a total BOD exceeding about 9 times for the one with 3 mg/L NH_4_^+^) and 10 times for the one with 6 mg/L NH_4_^+^ due to the low concentration of the dissolved oxygen in the river water models. Because of this, the nitrite ion concentrations in the 20 mg/L DEA model samples are relatively low compared to the reference sample of 3 mg/L NH_4_^+^ and much lower in the 6 mg/L NH_4_^+^ sample. These data are related to the samples, in which nitrogen reaches only the NO_2_^−^ form. If the oxidation state of nitrogen goes up to NO_3_^−^, oxygen consumption increases. However, in samples with 20 mg/L DEA due to oxygen deficiency, nitrate ions are reduced within redox processes.

Remarkably, the behavior of the NH_4_^+^ in the presence of DEA during the first days of the experiment follows a similar path as in the presence of 1-NA. In fact, during the first day the NH_4_^+^ content declines and then on the second day its concentration increases considerably.

For the 20.0 mg/L DEA containing sample, the difference between the ammonium nitrogen concentration values on 10–13 days and 20–42 days of the experiment is about 3.5 mg/L. Note that the maximum amount of nitrogen, about 3.8 mg/L, obtained at the decomposition of 20 mg of DEA would be comparable with just abovementioned difference. Seemingly, at the concentration of 20 mg/L DEA added in natural water samples, the increase of ammonium nitrogen within the interval of 20–42 days of the experiment is commensurable with the sum of the nitrogen amount resulted in various natural processes, its accumulation at the complete decomposition of DEA (between 20–42 and 10–13 days). For the experiment with 2 mg/L DEA, the decrease of NH_4_^+^ is concomitantly accompanied by the formation of NO_2_^−^, as in the reference sample. However, for the samples containing 20 mg/L of DEA, the decrease of NH_4_^+^ concentration and the increase of NO_2_^−^ concentration are delayed in the samples for both 3 mg/L and 6 mg/L of NH_4_^+^ initial concentrations (Fig. 5). It is possible that in these samples the oxidation of organic carbon takes place by the participation of oxygen from nitrite and nitrate ions due to a relative oxygen deficit in aquatic samples. There is a definite shortage of oxygen, as evidenced by decreasing the amount of NO_2_^−^ in the first ten days of experiments. Interestingly, the NO_2_^−^ concentrations in the sample for 6 mg/L of NH_4_^+^ initial concentration with 20.0 mg/L of DEA become high only after 50 days of the experiment (Fig. 5).

**Figure 6:**
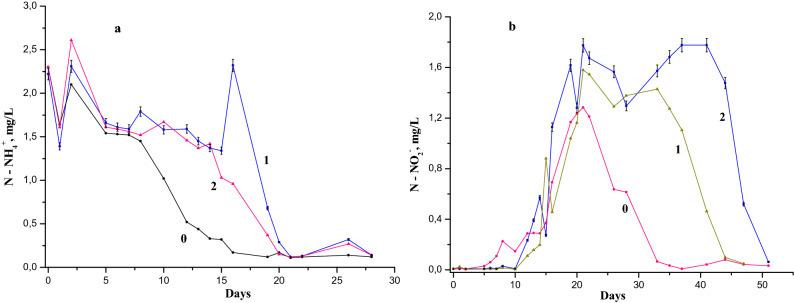
The dynamics of (**a**) ammonium (0—reference sample, 1—with 0.5 mg/L, 2—with 0.05 mg/L of diphenylamine) and (**b**) nitrite ions (0—reference sample, 1—with 0.05 mg/L, 2—with 0.5 mg/L, of diphenylamine) in model samples with 3 mg/L NH_4_^+^ in the Nistru River water.

**Figure 7:**
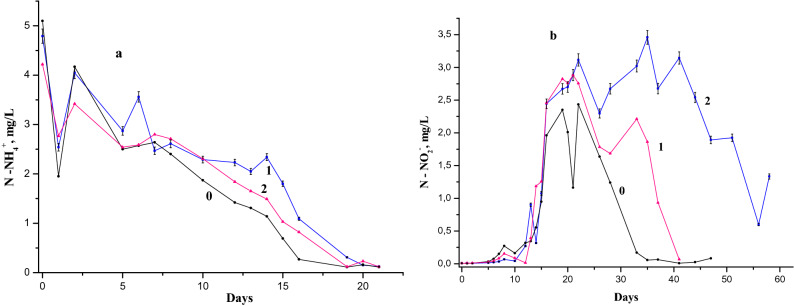
The dynamics of (**a**) ammonium (0- reference sample, 1- with 0.05 mg/L, 2- with 0.5 mg/L of diphenylamine) and (**b**) nitrite ions (0- reference sample, 1- with 0.05 mg/L, 2- with 0.5 mg/L of diphenylamine) in model samples with 6 mg/L NH_4_^+^ in the Nistru River water.

The industrial waste product in natural waters is often diphenylamine (DPA). The models of river water containing initially both 0.05 mg/L and 0.5 mg/L DPA (Figs. 6 and 7) have shown the effect of breaking oxidation of the NH_4_^+^ and NO_2_^−^. In contrast to 1-NA models of river water samples, the models of DPA have little influence on its concentration on the NH_4_^+^oxidation process. In the sample containing 0.5 mg/L DPA (curve 2 in Fig. 6) the oxidation of NH_4_^+^ is not slower than that in the samples with 0.05 mg/L DPA (curve 1 of Fig. 6), as it could be expected.

For natural water models with initial concentration of 3 mg/L NH_4_^+^, the difference between the samples with 0.5 mg/L and 0.05 mg/L DPA is completely surprising due to the increase in the NH_4_^+^ concentration on the 16th day. Most probably, the shift from 0.05 mg/L to 0.5 mg/L of DPA leads to the change of the oxidation dynamics of NH_4_^+^ for these two samples. The confirmation of this phenomenon is detailed in Table 2 (see the modifications from the days 14-16th to 19th day). During the period where the NH_4_^+^ oxidation dynamics has noticeable changes, each of the nitrogen species NH_4_^+^, NO_2_^−^ and NO_3_^−^ has a different behavior in each of the model samples (Table 2). In the sample containing 3 mg/L of NH_4_^+^ and of 0.05 mg/L DPA initial concentration, after the 14th and 15th days there is an increase of NH_4_^+^ concentration, while the NO_2_^−^ concentration is decreasing, and the NO_3_^−^ concentration is relatively constant (Fig. 6 and Table 2). The concentration of total soluble nitrogen (TSN) on the day 16th exceeds by about 1 mg/L this index for the days 14th, 15th and 19th. This difference is similar to the increase of NH_4_^+^ concentration compared with the previous days (14th, 15th). Due to the increase in the concentration of NH_4_^+^, there is a delay in its oxidation dynamics in the sample with 3 mg/L of initial concentration and 0.05 mg/L DPA compared to the sample with the same NH_4_^+^ concentration and 0.5 mg/L DPA.

**Table2:** Soluble mineral nitrogen in river water models within 14-19th days.

Test time, days	NH_4_^+^ nitrogen, mg/L	NO_2_^−^nitrogen, mg/L	NO_3_^−^nitrogen, mg/L	Total nitrogen, mg/L
**Initially 3 mg/L NH**_**4**_** and 0.05 mg/L DPA**				
14	1.37 ± 0.147	0.197 ± 0.013	2.43 ± 0.163	3.74 ± 0.187
15	1.34 ± 0.144	0.88 ± 0.057	1.52 ± 0.115	4.71 ± 0.2355
16	2.32 ± 0.249	0.457 ± 0.030	1.93 ± 0.141	3.49 ± 0.1745
19	0.68 ± 0.073	1.04 ± 0.067	1.77 ± 0.133	3.74 ± 0.187
**Initially 6 mg/L NH**_**4**_** and 0.05 mg/L DPA**				
14	1.49 ± 0.160	1.185 ± 0.077	4.62 ± 0.346	7.29 ± 0.3645
15	1.03 ± 0.111	1.257 ± 0.081	0.7 ± 0.052	2.99 ± 0.1495
16	0.82 ± 0.088	2.459 ± 0.159	7.47 ± 0.561	10.75 ± 0.5375
19	0.12 ± 0.013	2.824 ± 0.182	9.67 ± 0.725	12.61 ± 0.6305
**Initially 3 mg/L NH**_**4**_** and 0.5 mg/L DPA**				
14	1.42 ± 0.144	0.571 ± 0.0368	1.99 ± 0.149	3.98 ± 0.199
15	1.42 ± 0.152	0.274 ± 0.0177	2.92 ± 0.217	4.61 ± 0.2305
16	1.03 ± 0.111	1.129 ± 0.0728	3.29 ± 0.248	5.44 ± 0.272
19	0.93 ± 0.100	1.617 ± 0.1043	4.38 ± 0.328	6.93 ± 0.3465
**Initially 6 mg/L NH**_**4**_** and 0.5 mg/L DPA**				
14	2.34 ± 0.252	0.317 ± 0.0205	4.45 ± 0.334	7.11 ± 0.3555
15	1.8 ± 0.194	1.074 ± 0.0693	7.39 ± 0.545	10.16 ± 0.508
16	1.09 ± 0.194	2.445 ± 0.1577	8.48 ± 0.636	12.01 ± 0.6005
19	0.31 ± 0.0033	2.671 ± 0.1723	8.56 ± 0.642	11.54 ± 0.577

In the model containing 6 mg/L of NH_4_^+^ and 0.05 mg/L DPA, the contribution to TSN is the largest from the account of N–NO_2_^−^ and nitrogen nitrate. On the day 16th TSN has a maximum compared to the days 14th, 15th and 19th. In samples containing 3 and 6 mg/L NH_4_^+^ and 0.5 mg/L of DPA, the contribution to TSN decreases at the excess of nitrite and especially of nitrate. The formation and oxidation dynamics of NO_2_^−^ in the samples with 3 mg/L and 6 mg/L of NH_4_^+^ show several differences (Fig. 6 and 7). However, it can also be assumed some similarity of the dynamics in nitrite ion concentrations for these samples.

Comparing the influence of 1-NA and DPA concentrations on redox processes affecting on the nitrogen species in natural waters, one can conclude that the impact of the presence of 1-NA in natural waters is much higher. This is connected to the environmental safety and the requirement to develop new methods of decomposition, degradation/biodegradation of ammonium derivatives, containing benzene ring and naphthylamines^62–67^. Unfortunately, even if there are numerous methods developed for conversion of aniline and diphenylamine with a satisfactory result, the existing methods of decomposition or transformation for 1-NA are scarce, with an uncertain efficiency of decomposition and degradation/biodegradation.

## Conclusion

After the addition of an amount of ammonium ions to the clear water of the river, the absorption of NH_4_^+^ took place during the first day after initiation and the next day there is a desorption of ammonium ions. The impact of increasing amount of 1-NA, which is a harmful and mutagenic substance, in river water on the diminishing of the NH_4_^+^ sorption effect, shows that the nature of this absorption–desorption can be assumed to be biotic. Two types of supposed N-fixations were distinguished: (1) Ammonium absorption–desorption, e.g. the micro biotic reaction to ammonium (MBRA), at the instantaneous increase in the NH_4_^+^ concentration in the river waters (so-called shock/stress effect); (2) The NH_4_^+^ increase stimulated by a certain concentration, 0.05 mg/L of 1-NA. A noticeable effect of the attenuation of nitrification processes caused by the presence in the samples of ammonium derivatives was found. The simulation experiments with river water samples show that ammonium synthetic derivatives (1-NA and DPA) at concentrations below and equal to MAC have a impact of delayed on redox processes between NH_4_^+^, NO_2_^−^ and NO_3_^−^. The oxidation dynamics of NH_4_^+^ are slowed for first to second day at concentrations below or at MAC 1-NA. DPA to concentration equal at the MAC levels has a relative lower impact compared to 1-NA. As a rule, in similar model samples, the rate of ammonia oxidation in those with 6 mg/L NH_4_^+^ slightly exceeds those with 3 mg/L NH_4_^+^. The increasing of the concentrations of 1-NA and DPA causes the much noticeable effect of suppression on biochemical oxidation of ammonium and especially of nitrite ions. As the concentration of 1-NA and DPA increased to their 10 MAC, the delay time for NH_4_^+^oxidation reached approximately 5 days for the DPA samples and 10 days for the 1-NA samples. The oxidation time of the nitrite ion in the presence of 1-NA below and equal to MAC was about 4 days bigger compared to the reference sample. With the increase of this ammonium derivative the delay progresses and reaches for 10 1-NA MACs of about 35 days. For samples with 1-NA and 3 mg/L NH_4_^+^ or 6 mg/L NH_4_^+^ the differences were not significant in the NO_2_^−^ oxidation delay. For the DPA samples the delay compared to the reference sample was the following: (1) In the samples with the initial 3 mg/L NH_4_^+^ for the one with 1 DPA MAC the delay was equal to about 10 days and for one with 10 DPA MACs the delay was equal to about 18 days; (2) In the samples with 6 mg/L NH_4_^+^ for the one with 1 DPA MAC the delay was approximately 6 days and for the one with 10 DPA MACs the delay was approximately equal to 25 days. Experiments with diethylamine revealed an increase in NH_4_^+^ concentration due to its decomposition (approx. 3.5–3.8 mg/L nitrogen of NH_4_^+^). Diethylamine, being easily degradable, caused an increase in the concentration of ammonium ions due to the transformation of its amine nitrogen and the reduction of nitrites and nitrates through its organic carbon. Before the 50th day, in the samples, which contain 6 mg/L NH_4_^+^ and 20 mg/L DEA, the nitrite concentrations have lower values compared to the others, which could serve as an indicator of the oxygen deficit in this period of time.

## Data Availability

All data are included in the manuscript.

## Abbreviations

SAS-Ct/An: Cationic/anionic surfactants (surface-active substances)
1-NA: 1- Naphthylamine
DEA: Diethylamine
DNRA: Type of dissimilatory nitrate reduction to ammonium
DPA: Diphenylamine
MAC: Maximum allowable concentration
MBRA: Micro biotic reaction to ammonium
TSN: Total soluble nitrogen
COD: Chemical oxygen demand
BOD_5_: Biochemical oxygen demand in five days
